# Phylogeny-Based Comparative Methods Question the Adaptive Nature of Sporophytic Specializations in Mosses

**DOI:** 10.1371/journal.pone.0048268

**Published:** 2012-10-30

**Authors:** Sanna Huttunen, Sanna Olsson, Volker Buchbender, Johannes Enroth, Lars Hedenäs, Dietmar Quandt

**Affiliations:** 1 Department of Biology, University of Turku, Turku, Finland; 2 Department of Agricultural Sciences, University of Helsinki, Helsinki, Finland; 3 Nees-Institute for Biodiversity of Plants, University of Bonn, Bonn, Germany; 4 Department of Biosciences and Botanical Museum, University of Helsinki, Helsinki, Finland; 5 Department of Cryptogamic Botany, Swedish Museum of Natural History, Stockholm, Sweden; University of Umeå, Sweden

## Abstract

Adaptive evolution has often been proposed to explain correlations between habitats and certain phenotypes. In mosses, a high frequency of species with specialized sporophytic traits in exposed or epiphytic habitats was, already 100 years ago, suggested as due to adaptation. We tested this hypothesis by contrasting phylogenetic and morphological data from two moss families, Neckeraceae and Lembophyllaceae, both of which show parallel shifts to a specialized morphology and to exposed epiphytic or epilithic habitats. Phylogeny-based tests for correlated evolution revealed that evolution of four sporophytic traits is correlated with a habitat shift. For three of them, evolutionary rates of dual character-state changes suggest that habitat shifts appear prior to changes in morphology. This suggests that they could have evolved as adaptations to new habitats. Regarding the fourth correlated trait the specialized morphology had already evolved before the habitat shift. In addition, several other specialized “epiphytic” traits show no correlation with a habitat shift. Besides adaptive diversification, other processes thus also affect the match between phenotype and environment. Several potential factors such as complex genetic and developmental pathways yielding the same phenotypes, differences in strength of selection, or constraints in phenotypic evolution may lead to an inability of phylogeny-based comparative methods to detect potential adaptations.

## Introduction

Since Darwin’s *Origin of Species* [1], correlations between ecology and morphological traits in organisms have led biologists to postulate that adaptive diversifications are the driving force for morphological evolution. Speculations on the adaptive evolution of morphological traits still tend to be common, especially in papers dealing with evolutionary history and morphological evolution. Based on field observations of covariation between phenotypes and environments, for example, xerophytic plants in a Mediterranean-type vegetation, succulent plants in arid environments, and many specialized morphological structures in aquatic plants are called classical examples of true adaptations in many botany text books, because these traits may aid survival in the respective environments [2], [3]. Only rarely, however, evidence emerges on evolutionary processes that result in the phenotypes. For traits that have evolved in response to environmental selection pressure, the shift in ecology should take place before the shift in phenotype, but usually the evolutionary order between these shifts is unknown (see, however [4], [5]). Thus, as long as it is unclear if natural selection by the habitat is the driving force behind the evolution of observed traits, it is questionable whether these are adaptations in the strictest sense [6].

Testing the hypothesis of adaptive diversifications across a wide taxonomic scale may be rather challenging. Studies dealing with the origin of the adaptations are most often restricted to showing adaptive evolution within or between populations or, sometimes, between two or very few species. Most genetic methods commonly used for detecting adaptive evolution are non-applicable or will require rather extensive research efforts if a group with potentially adaptive traits involves a large number of species scattered among taxonomically diverse groups. However, phylogenetic approaches utilizing molecular phylogenies and information on distribution of traits among terminals allow detection of correlated evolution between ecology and morphological traits [7]–[10]. Correlated evolution of ecological and morphological shifts often appears as evidence for adaptive evolution [11]–[13]. Correlated evolution alone, however, does not directly confirm natural selection or fitness differences between the phenotypes, key factors that are needed for adaptive evolution. Phylogenetic approaches can, however, reveal potential adaptations by showing correlated evolution and a relative order of evolutionary changes in ecology and phenotypes. They can thus serve to point out a potential evolutionary link between a change in environment and a shift in phenotype and to detect whether the change in environment was followed by a shift in morphology.

Mosses growing as epiphytes on other plants form a taxonomically diverse group including species from most major lineages among the division Bryophyta. Epiphytes are especially common and are scattered among almost all families of pleurocarpous mosses, the crown clade of the subphylum including about half of the total 10,000 moss species in the world [14], [15]. The pleurocarpous mosses comprise typically perennial mosses with creeping stems and sporophyte-producing lateral branches; various pleurocarpous moss lineages have also repeatedly and independently conquered epiphytic habitats [16]–[20]. The major radiation among the group took place more than 165 to131 mya ago, during the late Jurassic and Cretaceous [21]. One hypothesis is that at least one of the driving forces behind the major pleurocarp radiation is the evolution of epiphytic life forms, especially on woody angiosperms [16], [22], [23]. This time-frame of pleurocarp evolution coincides with the radiation of angiosperms [24].

**Figure 1:**
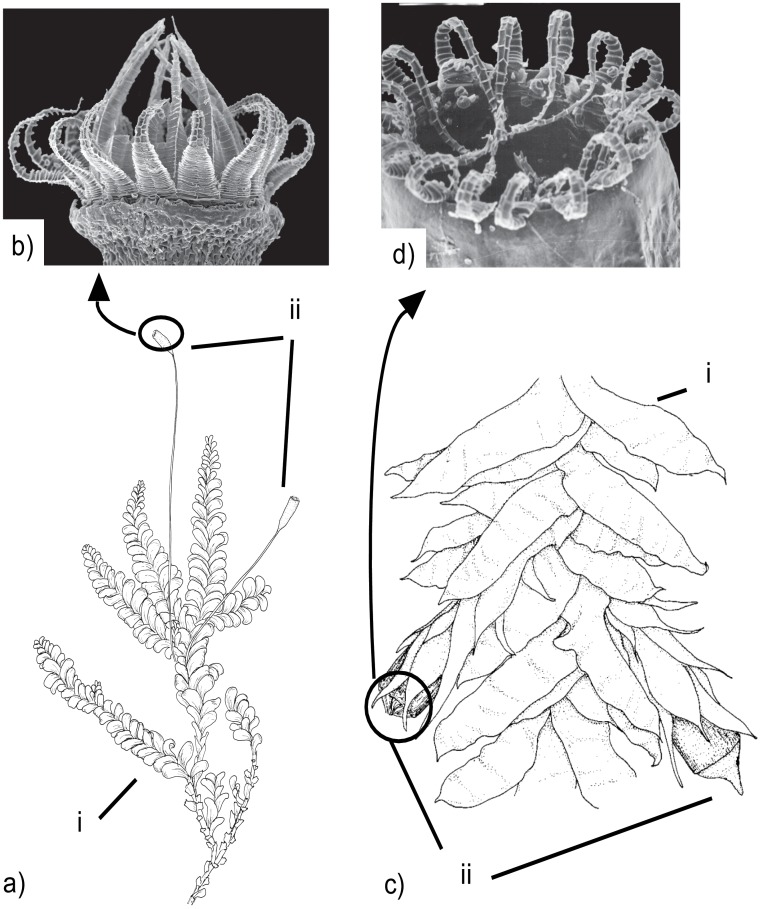
Sporophyte structure in Neckeraceae. Example of a perfect and a specialized sporophyte structure in Neckeraceae. a) *Homalia trichomanoides* gametophyte (i) and sporophytes (ii) with long setas and slightly inclined capsules; b) SEM view of well-developed hypnalean peristome in *H. trichomanoides*. c) *Neckera pennata* gametophyte (i) and sporophytes (ii) that have short setas immersed in perichaetial leaves and upright capsules; d) SEM view of reduced peristome in *N. pennata*. Pictures reprinted with permission of M. S. Ignatov and E. Ignatova.

Despite their diverse origin, epiphytic mosses tend to share certain morphological characteristics. In particular, their sporophytes are often reduced to various degrees: the seta that carries the spore capsule is short, the capsule is orthotropous, making the capsule horizontal on vertical substrates, the peristome that regulates spore dispersal at the mouth of the capsule is reduced and is capable of only weak hygroscopic movement (Fig. 1). The sporophyte reductions seem to be linked with xerophytic habitats [25], [26], and epiphytes grow in a special form of xerophytic habitat. Drought in epiphytic habitats has also been assumed to result in trade-offs in evolution of sexual systems and life history traits in epiphytic lineages of the liverwort genus *Radula* [27]. As early as 1908, Grout [28] observed in mosses an association between these specialized sporophytic characters and the epiphytic habitat and suggested this phenomenon to be an adaptation. The specializations appear indeed to be very common and easy to find among pleurocarps. For example, among the 439 pleurocarpous moss species studied by Hedenäs [26], some 10% of species with capsules have an erect capsule and a peristome with some reduced traits. As sporophyte structures are responsible for producing and dispersing spores, changes in a sporophyte may also impact strongly upon fitness. A plausible explanation for the repeated evolution of similar morphological traits under similar environmental conditions is therefore that they are beneficial for the survival of individuals in those environments.

We aim to test here whether evolution of morphological specialization in mosses is correlated with their shifts into exposed or epiphytic habitats. We use data from the pleurocarpous moss families Neckeraceae and Lembophyllaceae that both show several independent transitions to epiphytic habitats and a considerable degree of variation in their sporophytic traits [20]. Based on a connection that was noted in earlier studies [17], [19], [20], [25], [26], [28], we selected for further analysis eight candidate traits. Their evolution seemed to be connected with a shift to epiphytic or exposed habitats, and we tested correlated evolution between the traits and a habitat shift using a Bayesian approach [10]. We tested the order of the character-state changes for morphology and habitat shifts by contrasting ancestral state reconstructions and by comparing the fit of two evolutionary models that had different transition rates for dual character states. Based on the results, we will distinguish morphological specializations that are potentially adaptations to epiphytic and exposed habitats, discuss other possible explanations for convergent evolution, and evaluate the utility of this method in detecting adaptive evolution in general.

## Results

### Ancestral State Reconstructions for Habitat Shifts and Morphological Traits

**Figure 2:**
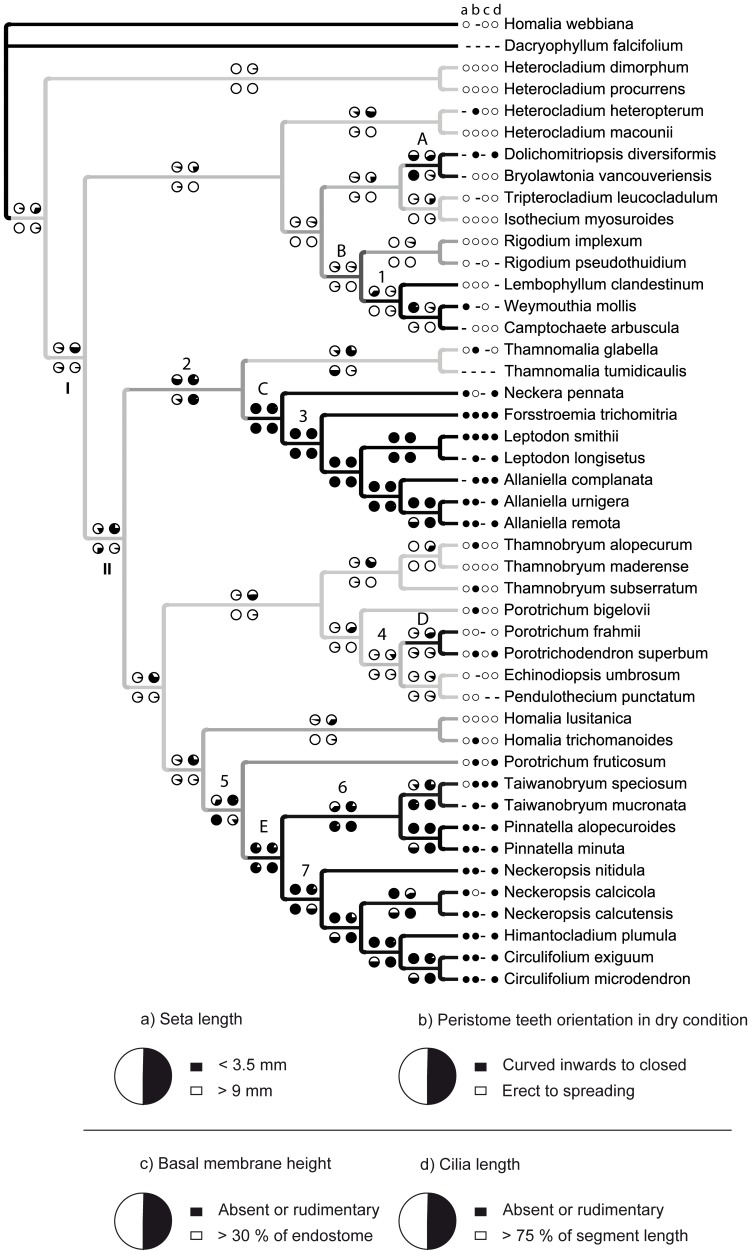
Ancestral character state reconstruction for habitat preference and four morphological traits. Ancestral character state reconstruction for habitat preference and four morphological traits that evolution may correlate with the habitat shifts among Neckeraceae and Lembophyllaceae. The color of the branches in the inferred Bayesian topology represents two states of the habitat: on soil/unexposed (light gray) and epiphytic/exposed (for branches with probability >0.95 = black). Branches with probability >0.90 but <0.95 for epiphytic/exposed habitats are with dark gray color. Probabilities for morphological ancestral character state are shown as pie diagrams in the nodes. BayesFactor (BF) support for epiphytic/exposed habitat preference is shown below branches. For morphological traits BF for a derived character state is indicated with color of pie diagrams: BF <2 light gray, BF >2 dark gray; and BF >5 with black (see Table 1). Pie diagrams along branches are in the same order as in the legend showing their character states (a–d). Character states for terminals are stated before the taxon name. Dash (−) indicates missing or inapplicable data. Nodes A–E with show lineages with shifts to epiphytic or other exposed habitats.

Ancestral state reconstructions favored with high probability a scenario that the ancestor of the Lembophyllaceae – Neckeraceae clade (node I, Fig. 2) as well as the ancestor of all Neckeraceae species (node II, Fig. 2) lived on soil or in an unexposed habitat (Fig. 2, Supporting information, Appendix S2). These ancestors had higher probabilities for plesiomorphic character states for seven morphological traits of the total eight studied. Only character 2 (c2), the operculum shape, showed a character-state shift between nodes I and II (Fig. 2, Supporting information, Appendix S2; see Supporting information, Appendix S1 for a list of all characters). The ancestor of the Lembophyllaceae – Neckeraceae clade thus had a conical to rostrate operculum, i.e a plesiomorphic state, whereas the ancestor of all Neckeraceae species had a derived state with a long-rostrate operculum (Fig. 2, Supporting information, Appendix S2).

**Table 1:** Bayes Factor (BF) support for four morphological traits and habitat preference.

node (Fig. 2)	node(Fig. S1)	Habitat preference			3) Dry peristome			5) Basal membrane			6) Cilia			8) Seta length		
		P(0)	P(1)	BF (1)	P(0)	P(1)	BF (1)	P(0)	P(1)	BF (1)	P(0)	P(1)	BF (1)	P(0)	P(1)	BF (1)
I	5	−25.44	−29.03	−7.17	−23.95	−25.57	−3.24	−8.53	−13.44	−9.81	−15.62	−21.93	−12.61	−15.44	−20.25	−9.61
II	16	−25.51	−28.45	−5.87	−24.61	−24.18	0.86	−8.34	−12.79	−8.91	−15.26	−20.96	−11.40	−15.36	−19.48	−8.23
A	10	−26.39	−26.04	0.71	–	–	–	–	–	–	−20.31	−15.79	**9.05**	−14.97	−18.05	−6.17
B	14	−28.37	−25.52	**5.70**	–	–	–	–	–	–	–	–	–	–	–	–
	15	−29.55	−25.37	**8.36**	–	–	–	–	–	–	–	–	–	−15.48	−16.63	−2.29
	17	−25.52	−25.76	−0.48	−25.12	−24.22	1.81	−9.11	−9.48	−0.73	–	–	–	−15.19	−17.73	−5.08
C	20	−27.67	−25.72	**3.89**	−26.84	−23.76	**6.16**	−12.05	−8.53	**7.03**	−18.23	−16.02	**4.43**	−18.04	−15.32	**5.44**
	21	−29.87	−25.56	**8.63**	−27.55	−23.77	**7.57**	−13.97	−8.80	**10.34**	−21.19	−15.59	**11.20**	−20.02	−15.09	**9.86**
	22	−30.30	−25.75	**9.11**	−28.35	−24.12	**8.45**	−14.58	−8.42	**12.32**	−22.07	−15.44	**13.26**	−21.01	−15.44	**11.14**
	23	−32.75	−25.47	**14.56**	−29.94	−23.91	**12.07**	−14.44	−8.55	**11.78**	−25.41	−15.80	**19.22**	−21.21	−15.01	**12.41**
	24	−31.41	−25.46	**11.91**	−29.19	−24.09	**10.21**	−14.05	−8.50	**11.09**	−23.91	−15.44	**16.94**	−21.42	−15.08	**12.67**
	25	−31.85	−25.48	**12.75**	−28.92	−24.22	**9.40**	−13.08	−8.22	**9.72**	−24.53	−15.46	**18.13**	−22.28	−15.08	**14.41**
D	32	−26.96	−25.61	**2.71**	–	–	**–**	–	–	–	–	–	–	–	–	–
	35	−25.48	−25.54	−0.12	−26.07	−23.99	**4.16**	−8.67	−12.96	−8.59	−17.91	−15.63	**4.57**	–	–	–
E	36	−25.64	−26.95	−2.62	−25.99	−23.62	−4.75	−8.62	−10.57	−3.91	−18.81	−15.39	**6.85**	−15.31	−17.18	−3.73
	37	−26.66	−25.78	1.75	−26.49	−24.16	**4.66**	−9.21	−9.25	−0.08	−20.33	−15.38	**9.89**	–	–	–
	38	−25.93	−26.56	−1.26	−26.30	−23.66	**5.27**	−8.93	−10.63	−3.40	−21.08	−15.60	**10.94**	−17.77	−15.31	**4.90**
	39	−27.32	−25.73	**3.18**	−26.13	−24.24	**3.79**	−9.61	−9.32	0.58	−20.87	−15.94	**9.84**	–	–	–
	40	−30.14	−25.44	**9.40**	−28.28	−24.13	**8.30**	−8.87	−9.29	−0.84	−23.52	−15.52	**15.99**	−19.67	−15.01	**9.32**
	41	−25.71	−26.47	−1.51	−26.18	−24.82	**2.72**	−8.78	−10.07	−2.58	−22.48	−15.38	**14.20**	−19.68	−15.12	**9.13**
	42	−25.71	−27.39	−3.37	−25.55	−23.69	**3.71**	−8.51	−10.42	−3.82	−22.69	−15.53	**14.32**	−20.41	−15.28	**10.27**
	43	−27.22	−25.57	**3.30**	−26.95	−23.97	**5.97**	−9.08	−9.81	−1.46	−23.39	−15.51	**15.76**	−21.03	−15.16	**11.74**
	44	−27.54	−25.65	**3.78**	−26.89	−23.86	**6.06**	−8.66	−9.95	−2.57	−23.36	−15.64	**15.44**	−21.26	−15.12	**12.26**

Bayes Factor (BF) support for ancestral states that earned higher probabilities at the nodes with probability >0.9 for a derived state and nodes I and II for four morphological traits and habitat preference. BFs are based on difference in harmonic means of likelihoods derived from two analyses, where character state at a given node is constrained to be either 0 or 1. BF >2 is considered as positive evidence and BF >5 as strong support for the character state’s gaining the higher likelihood at the node. For character descriptions and coding of characters states and nodes see Fig. 2, Supporting information, Appendix S1, and Fig. S1. Probabilities for derived character states at each node are in the Supporting information Appendix S2 and in Fig. 2.

Shifts to exposed epilithic or epiphytic habitats have occurred in five lineages (Fig. 2; Table 1, Supporting information, Appendix S2): 1) in the *Dolichomitriopsis diversifolia* - *Bryolawtonia vancouveriensis* clade (Lembophyllaceae, node A), 2) in the core Lembophyllaceae (node B), 3) in the *Neckera* clade (Neckeraceae, node C), 4) in the *Porotrichum frahmii* – *Porotrichodendron superbum* clade (Neckeraceae, node D), and 5) in the *Pinnatella* clade (Neckeraceae, node E). Despite higher probabilities for habitats on exposed or epiphytic substrates, Bayes Factors (BFs) lend positive support (BF >2) to a derived character state only at nodes B, C, D and two lineages within the *Pinnatella* clade (Fig. 2, Table 1). For none of the morphological traits studied did transitions to derived states appear always in the same nodes along with a shift in habitat (Fig. 2; Supporting information, Appendix S2).

### Correlated Evolution between Habitat Shift and Morphology

For four morphological traits, Reversible Jumping Markov Chain Monte Carlo (RJ MCMC) applying a dependent model (D; a model where morphological and habitat evolution are dependent on each other), the harmonic mean of log-likelihood scores sampled during the chain was significantly higher than for the chain with an independent model (I; a model where morphology and habitat evolve independently) (Table 2). These four traits were peristome orientation in a dry condition (c3), height of endostomial basal membrane (c5), and endostomial cilia (c6), and seta length (c8; Table 2). BF strongly favored the D model of evolution for these (BF >5; Table 2). The D models were also visited during the chain more frequently than expected (Table 2), which also supported their better fit to the data. For two morphological traits: peristome orientation in a dry condition and endostomial cilia, I models were not visited at all during the best RJ MCMC, lending the strongest possible support for correlated evolution of morphology and habitat preferences (Table 2).

**Table 2:** Correlated evolution between change in morphology and shift to exposed epiphytic or epilithic habitat.

								Number of I visits						
Character	D mean lnL	D st.dev. lnL	D max lnL	I mean lnL	I st.dev. lnL	I max lnL	BF	D1	D2	D3	posterior odds		posterior/prior odds	BF
1) Post fertilization growth of perichaetial leaves	−43.94	0.33	−43.65	−43.76	0.06	−43.70	0.10	18	22	**25**	799.00		1.93	1.32
2) Operculum shape	−45.02	0.36	−44.61	−44.36	0.57	−43.97	−1.27	34	36	**30**	665.67		1.61	0.95
**3) Dry peristome**	−46.57	0.18	−46.43	−49.38	0.09	−49.31	**5.75**	**0**	3	6	NA		NA	**NA**
4) Spore size	−51.85	0.12	−51.72	−52.07	0.02	−52.06	0.67	**14**	15	10	1 427.57		3.45	2.48
**5) Basal membrane**	−32.14	0.20	−32.00	−34.79	0.20	−34.63	**5.26**	5	**1**	0	19 999.00		48.35	**7.76**
**6) Cilia**	−40.97	1.47	−40.02	−42.68	0.14	−42.55	**5.07**	**0**	1	3	NA		NA	**NA**
7) Peristome	−37.95	0.13	−37.80	−35.90	0.08	−35.81	3.98	1	**4**	4	4 999.00		12.09	4.98
**8) Seta length**	−38.42	0.14	−38.30	−41.58	0.13	−41.44	**6.28**	**1**	2	2	19 999.00		48.35	**7.76**

Results from test of correlated evolution between change in morphology and shift to exposed epiphytic or epilithic habitat. Test result based on i) comparisons of harmonic means of likelihoods (lnL) from reversible-jump Markov Chain Monte Carlo (RJ MCMC) runs with an independent (I) and a dependent (D) model of character evolution; and ii) numbers of visits in I models during RJ MCMC runs (D1, D2, D3). For i) mean of harmonic means (mean lnL), standard deviations (st.dev. lnL), and maximum harmonic mean of likelihood (max lnL) for three I and D runs are given. Bayes Factor values (BF) are calculated using the maximum harmonic mean of likelihood obtained from the best I and D run, i.e. the run yielding the highest likelihood after 200 000 000 iterations (I max lnL and D max lnL in the table). For ii) chains were run three times (D1, D2, D3), and for the best run, number of visits to I models was compared with the prior odds (see 10). BFs >5 based on prior and posterior odds give support for unexpectedly high number of visits to D models, and thus strongly support the evolutionary model assuming correlated evolution between morphological character change and habitat shift. When the support for the D model is the strongest and visits to I models are absent, zero values in the divisor yield non-applicable (NA) BF. BF >5 are considered strong evidence for correlated evolution [46], [48] and are bolded.

### Change First in Habitat or in Morphology?

In ancestral character state reconstructions, a shift to a derived morphological character state appears before the shift to exposed or epiphytic habitats at least in some lineages for all correlated traits (Fig. 2, Supporting information, Appendix S2). Derived morphology was frequently gained before habitat shift especially in nodes C and E. However, even if probabilities for derived states were already higher before reaching the nodes C and E, the BFs mostly lend support for shifts only at those nodes or even after (Fig. 2, Table 1; see e.g. c8). Derived character states were gained with at least positive BF support at the same node with shift in habitats or after the shift for the following traits: at node A, cilia length (c6); at nodes B and D, none of the correlated traits; at node C, dry peristome (c3), basal membrane height (c5), cilia length (c6), and seta length (c8); and at node E, dry peristome (c3), cilia length (c6), and seta length (c8) (Fig. 2, Table 1).

Rate coefficients for dual character state change indicated that the shift in habitats occurred before the change in morphology for three of the correlated traits, height of endostomial basal membrane (c5) and cilia (c6), and seta length (c8). For these, rate coefficients q12 (change in habitat preference but not in morphology) were significantly larger than q13 (change in morphology but not in habitat preference) (Table 3). For only one morphological trait, orientation of dry peristome (c3), q13 was smaller than q12 (Table 3). However, for all morphological traits, the fit of the evolutionary model where the rate coefficients were restricted as equal (q13 = q12, i.e. a model with seven parameters for dual character state transitions; R in Table 3) was not significantly worse than the model where rates were allowed to vary freely (a full model with eight parameters; Table 3). Differences between the rate coefficients were thus so small that they did not lead to a significant difference in harmonic means of log likelihoods from MCMC chains with the restricted model and the full eight-parameter model.

**Table 3:** Comparisons between rates of dual character state change in morphology and habitat.

Character	meanlnL D	maxlnL D	mean lnL R	maxlnL R	BF	q12	q13	P	Mann-Whitney U
3) Dry peristome	−46.96	−46.93	−47.13	−47.05	−0.18	55.23	**68.65**	**0.00**	133 000 000
stdev	0.03		0.09			23.13	21.23		
5) Basal membrane	−33.71	−33.60	−34.28	−33.72	−0.01	**65.39**	57.17	**0.00**	165 800 000
stdev	0.11		0.54			24.48	27.35		
6) Cilia	−40.63	−40.39	−41.36	−40.79	−0.79	**72.32**	45.66	**0.00**	92 495 436
stdev	0.27		0.78			20.81	27.89		
8) Seta length	−39.71	−39.63	−39.94	−39.87	−0.49	**66.29**	47.92	**0.00**	124 900 000
stdev	0.07		0.11			23.80	28.13		

Comparisons between rates of dual character state change in morphology and habitat. q12 is the rate coefficient for character change where morphology changes while habitat preference remains unchanged ([0,0]-> [0,1]), and q13 is the rate of the change where morphology remains unchanged while habitat changes ([0,0]-> [1,0]). Difference in rates was tested by running an MCMC chain applying the model of dependent evolution for morphological and habitat character state change (D; 8 parameters) and with the restricted model where q12 and q13 were forced to be the same (R; 7 parameters). Bayes Factors (BF) served to estimate whether the difference in likelihoods for R and D models was statistically significant. Both for R and D models, MCMC runs were repeated three times; means for D runs (mean lnL D) and for R runs (mean lnL R) are in the table. BF was calculated based on the best run, i.e. the one yielding the highest likelihood (max lnL D and max lnL R). BF >5 were regarded as strong support. Rate coefficients were also sampled during MCMC chains with a D model and used for testing the difference between q12 and q13. Means and standard deviations for the rate parameters (columns q12 and q13) from the run yielding the best likelihood are given and significance of differences between the rates is tested.

## Discussion

### Adaptive Evolution in Explaining the Match between Habitat and the Presence of Specialized Traits

Adaptation alone is not able to explain the convergent evolution of sporophytic specializations in mosses that grow in epiphytic and other exposed habitats. Only for four specialized sporophytic traits, the short seta and three traits of the peristome, shifts in phenotype were correlated with a habitat shift (Fig. 2). In accordance with differences between transition rates, the shift to the derived morphological character state occurred after the shift in the habitats; three of these, seta length and two endostomial traits, may possibly be adaptations in the strict sense (Fig. 2) [6], [29]. These three traits as well as the majority of other specialized traits are sporophytic reductions. As the function of alternative sporophytic phenotypes in different environments has not been explored, their effect on fitness and presumed role of natural selection will, however, remain untested. Besides direct impact of sporophytic reductions on dispersal and fitness, their evolution may be explained by an indirect increase in fitness via bionenergetics, because less biomass and energy need to be spent for sporophyte production. Habitats high above the ground can facilitate dispersal of spores, and thus eliminate, e.g., the need of a long seta as the whole plant body takes over its role. In contrast, strong stabilizing selection in sheltered low-elevation habitats in the forest-floor layer may favor retaining the long seta and capsules with a perfect peristome that actively disperses the spores by its hygroscopic movements [19], [25]. In epiphytic and other exposed habitats ecological constraints may be relaxed, and some of the complex sporophytic traits that have become unnecessary are reduced [30]. In general, loss or reduction of structures is considered to have a simpler genetic basis than their gaining [31], a fact which may favor parallel evolution.

Adaptive evolution could not explain correlated evolution between the orientation of dry peristome and habitat shift (Table 3), since a peristome in which the teeth are curved inwards to close the capsule in the dry state had already evolved before the shift in habitats. It could be a pre-adaptation, i.e. of an evolutionary origin of which is not necessarily linked to selection or higher fitness in the current environment (exaptation [6]). Evolution of some other morphological traits that bryologists have traditionally linked with a shift to epiphytism, such as capsule orientation, does not correlate at all with the habitat shift, but these have appeared before the shift (see also [20]).

### Other Processes Potentially Contributing to the Match between Morphology and Environment

Although a functional fit between organisms and their environment is often assumed to be due to adaptive evolution, ecological processes may also contribute significantly to the observed association between morphology and the environment [32]. Community assembly processes and habitat tracking, together with the higher fitness of the derived phenotype in exposed epiphytic and epilithic habitats, may explain the frequent occurrence of these traits in Lembophyllaceae and Neckeraceae species that grow in these habitats. Organisms with an already existing phenotype that fits better for a certain environment will thus be found more often in that habitat; this results in an observable fit between the specialized phenotypes and environments. After establishment in a new habitat, habitat selection and stabilizing selection will enable the creation and maintenance of the association [4].

If the evolutionary order of the shift in habitats versus phenotype goes unstudied, this may result in a false impression of the potential adaptive origin of the traits. For example, a low specific leaf area (SLA) and small leaves in flowering plants did not evolve as adaptations to dry Mediterranean climates. These features appeared in tropical forests that formed the ancestral vegetation in areas that nowadays host chaparral vegetation [4]. The parallel evolution of C_4_ photosynthesis in some grass lineages was often considered an adaptation to arid environments or to changing atmospheric CO_2_ concentrations, but the C_4_ phenotype evolved before the shift to arid habitats [5]. It could thus represent a pre-adaptation to arid habitats, where the increased frequency of the phenotype as well as positive selection of the genes behind it are undoubtedly due to better fitness in arid environments [5], [33]. The patterns observed in these two cases are thus analogous to some of the traits in epiphytic pleurocarpous mosses.

### Phylogeny-Based Comparative Methods in Recognizing Shared Adaptations

The ability of phylogeny-based comparative methods to successfully detect correlated evolution between ecological and morphological traits, and thus potential adaptations, is based on the assumption that the same environmental selection pressure leads to similar phenotypic changes in different lineages. This implies that, first, the selective pressures do not vary significantly among populations due to highly similar ecological conditions. Second, in the different taxa and lineages the underlying genetic and other mechanisms for adapting to environmental change must be fairly similar. Third, the selection should lead to similar changes in phenotype despite differing combination of the original character states across taxa.

However, once the evolution of shared adaptation does not follow the assumptions made in phylogeny-based comparative methods they would have been undetected in our study group (see also [29]). In the few cases where we could not detect a significant correlation between morphological and habitat shifts, an analysis of genes that regulate the evolution of specialized sporophytic traits for epiphytic mosses might probably find signs of selection in some lineages.

Neither of the above mentioned assumptions are necessarily true when adaptations are studied on the present macroevolutionary scale. The strength and direction of environmental pressures may vary between taxa due to the world-wide sampling, because a wide geographical scale leads to a wider variation in microhabitat quality. In addition, morphological or physiological trait complexes can be acquired via differing underlying genetic pathways in different lineages [33]–[38]. Recent results suggest a surprising number of alternative genetic and developmental pathways behind similar trait complexes in different lineages; this may be explained by variation in evolutionary patterns among groups [37], [39]–[41]. In mosses, slight differences in structure and trait combinations of specialized sporophytes between epiphytic lineages could indicate developmental or genetic differences in phenotype regulation. Due to the unique life cycle among land plants, with a dominant perennial gametophyte generation (see Fig. 1), gene expression and genetic regulation of sporophytic traits in mosses differ from those of derived land plants [42], [43]. The limited information on their functional genetics hampers further evaluation along these lines in bryophytes.

Finally, phenotypic, genetic and developmental constraints may either prevent or enhance the shift to the adapted phenotype and favor convergent evolution [37], [38], [40], [44]. Conflicting responses on selection in two traits in which genetic or developmental pathways are linked may constrain the changes in the phenotype [45]. Coevolution within character complexes and constraints that allow traits to shift to the adapted state only in combination with some other changes may explain why three of the four correlated traits in our study were endostomial traits.

### Three Promising Candidate Traits for Further Studies on Adaptive Evolution of Epiphytic Mosses

The phylogenetic approach that we apply here provides a simple and cost-effective way to test hypotheses regarding the evolution of morphological specializations in relation to the habitat. Three traits that are correlated with habitat shift, seta length and two endostomial traits, may be adaptations to epiphytic or other exposed habitats (Fig. 2). Adaptive evolution is, however, not the only process that explains a high frequency of some derived traits in those habitats [25], [26], [28], since several specializations did not evolve as a response to the habitat shift. Additional studies are also needed to confirm selection due to differential fitness of reduced and perfect sporophyte morphology in epiphytic or other exposed habitats versus the forest floor. Research on the evolution of adaptations and adaptive diversification are mostly limited to populations or lower taxonomic levels and their methods are often difficult to apply to macroevolutionary studies such as the ones detecting selection in distantly related taxa. Although recent advances on the background of adaptive and convergent evolution [41] suggest the phylogenetic approach may in some cases have limitations in pointing out potential adaptations, any positive result will still be useful for sorting out the most promising candidate traits [29]. Our results provide information on processes that contribute to ecological specialization on a taxonomic scale that is rarely explored and allow valuable insights into the mechanisms of diversification and evolution of differences among organisms. Both are central questions in biological research.

## Materials and Methods

### Phylogenetic and Ancestral Character State Reconstruction

In order to test correlated evolution and to reconstruct ancestral states with BayesTraits [46], we scored character states for eight morphological characters and habitat preference with binary coding (Supporting information, Appendix S1). Morphological traits were selected among larger selection of traits that were studied in our earlier study for their utility to delimit taxonomic groups in Lembophyllaceae and Neckeraceae [20]. For habitat preference species were coded as occurring in the habitat where it is most typically found. Some moss species, however, can be found in variety of different habitats in rendering the assignment of habitat preference difficult. Especially in the case of predominantly epiphytic and epilithic species decision between these two character states may be difficult [47]. The basis for coding was our field experience of the species in different parts of their distribution areas, the information given in the literature and local floras, as well as habitat information on herbarium labels. We calculated support for preferred ancestral states at critical nodes with a shift in character state with Bayes Factors using the “fossil” command in BayesTraits [46].

The molecular data, methods of phylogeny reconstruction and ancestral states to reconstruction with BayesTraits [46] were described in our earlier study that aimed at clarifying taxonomy of the group [20].

### Tests of Correlated Evolution

We performed tests of correlated evolution between habitat shifts and morphological traits using a Bayesian approach as implemented in BayesTraits [10]. The method utilizes a molecular phylogeny ([20]; Supporting information, Fig. S1) and distribution of morphological and habitat traits in terminals. It compares fit of two evolutionary models for two discrete characters, i.e. a model of correlated evolution (dependent evolution; D) employing up to eight rate parameters for dual character state transitions and an independent model of character evolution (I) with up to four rate parameters.

A reversible-jump Markov Chain Monte Carlo (RJ MCMC) served to sample trees and model parameters according to their posterior probabilities under the D and I models. Rate priors were set to vary within a uniform distribution between 0 and 100. We monitored acceptance rates and they were set to a rate deviation of approximately 20%. Each RJ MCMC was run for 1 000 000 000 generations and for all combinations of morphological character – habitat runs was repeated three times to check that log-likelihood values and harmonic means did not significantly differ between converted chains [46].

The fit of two competing models, I and D, was evaluated by two methods. First, RJ MCMC was run three times with both an independent (I) and a dependent (D) model of evolution. Support for either of the models was estimated by comparing harmonic means of likelihoods from I and D runs with logarithmic Bayes Factors (BFs). BF >5, based on one of the three D runs and the three I runs, was regarded as strong support for correlated evolution between a morphological trait and the habitat shift(s) [10]. The second approach was based on a property of the RJ MCMC that in the chain in which all eight dual character-state transitions can occur freely, the number of visits to the dependent or independent model is propositional to the posterior probability of the model [10]. Support for correlated evolution was thus also evaluated by comparing the ratio of prior and posterior odds for visits in I and D models during the chains [10]. Support for either model was estimated by use of BF.

### Change First in Habitat or in Morphology?

We detected the order of character change in the phylogeny by three different methods. First, we compared ancestral state reconstructions for habitat preference and morphology. Second, rate coefficients were sampled from one out of three RJ chains with the dependent model of evolution. We tested for difference in the posterior distribution of rate coefficients for change in morphology but not in habitat preference (q13; from [state for habitat preference = 0, state for morphology = 0] to [state for habitat preference = 0, state for morphology = 1]) and for change in habitat preference but not in morphology (q12). Rate coefficients for character change where morphology changes while habitat preference remains unchanged (q12; [0,0]-> [0,1]) were compared with rates of the change where morphology remains unchanged while the habitat changes (q13; [0,0]-> [1,0]). The statistical significance of the difference was tested by the non-parametric Mann-Whitney t-test. Third, evolutionary significance of difference in rate coefficients was confirmed by running an MCMC chain with the dependent model of evolution (eight rate parameters) and with restricted dependent model where q12 and q13 were set to equal [8]. Settings and method for running MCMC with BayesTraits were the same as above. The fit of these models was compared with BFs based on harmonic means of the posterior probability of likelihoods.

## Supporting Information

Figure S1 **Bayesian tree for moss families Neckeraceae and Lembophyllaceae.** Majority consensus of trees sampled after stationarity in the Bayesian analysis of the matrix including indels (for details, see [20] Olsson et al. 2009). Values along the branches indicate posterior probabilities (above the branches) and bootstrap support values from the parsimony analysis (below). The first value corresponds to the analyses with the matrix with insertion-deletion coding included in the analyses. Correlated evolution of habitat shift and morphological traits was tested for the subtree within a shaded box. Numbers indicate the nodes for which probabilities for derived ancestral character state are given in Supporting information Appendix S2.
(TIF)

Appendix S1 **Coding for habitat preferences and morphological character states.**
(DOC)

Appendix S2 **Ancestral character state reconstructions for evolution of eight morphological characters and habitat preferences in the moss families Neckeraceae and Lembophyllaceae.**
(DOC)
