# Supplementary figures and images for: Phylogeny-Based Comparative Methods Question the Adaptive Nature of Sporophytic Specializations in Mosses
Source: PLoS One. 2012 Oct 30;7(10):e48268. doi: 10.1371/journal.pone.0048268 (PMC3484137; doi:10.1371/journal.pone.0048268)

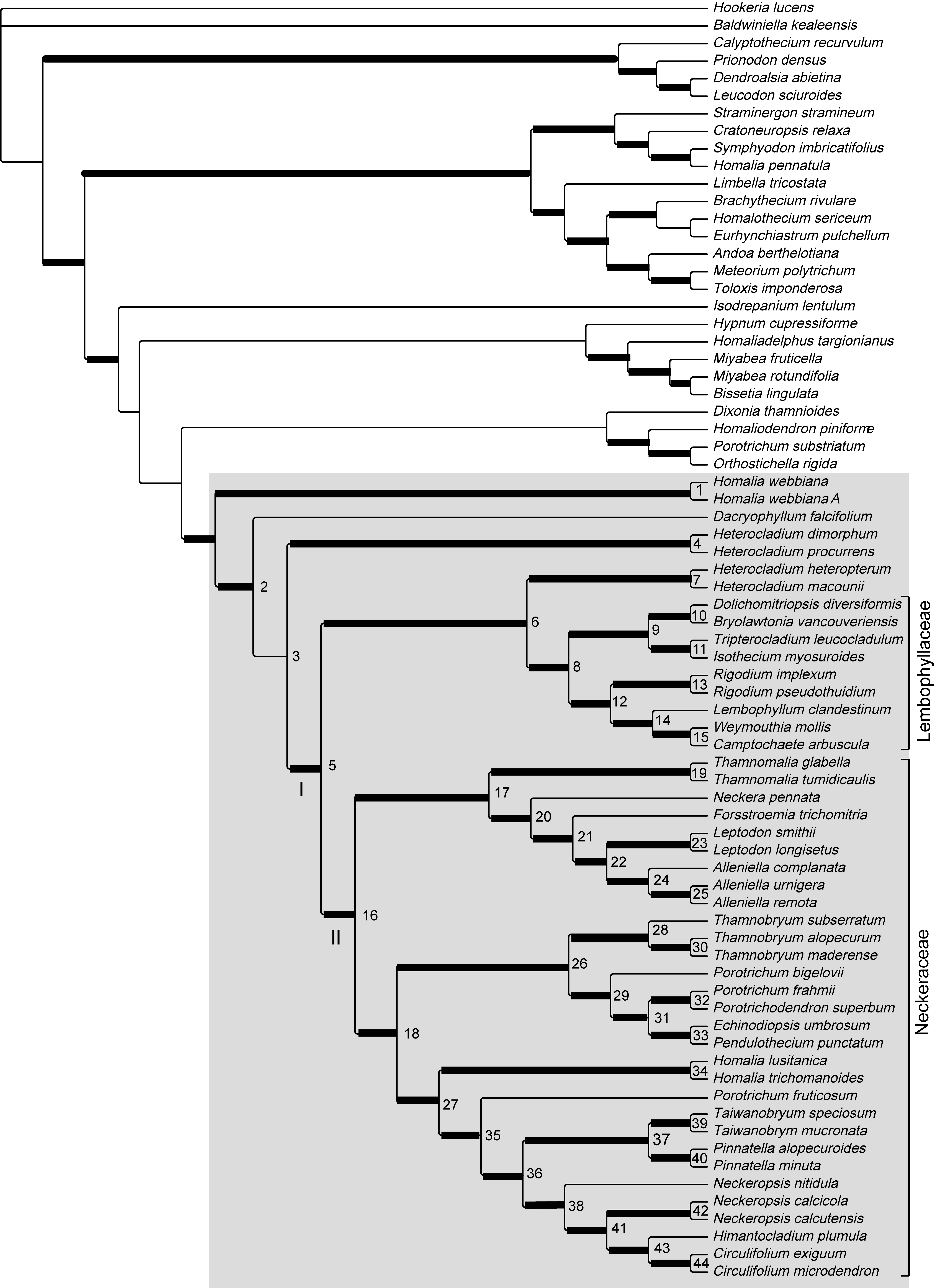

Supplement: Figure S1 — Bayesian tree for moss families Neckeraceae and Lembophyllaceae. Majority consensus of trees sampled after stationarity in the Bayesian analysis of the matrix including indels (for details, see [20] Olsson et al. 2009). Values along the branches indicate posterior probabilities (above the branches) and bootstrap support values from the parsimony analysis (below). The first value corresponds to the analyses with the matrix with insertion-deletion coding included in the analyses. Correlated evolution of habitat shift and morphological traits was tested for the subtree within a shaded box. Numbers indicate the nodes for which probabilities for derived ancestral character state are given in Supporting information Appendix S2. (TIF) [file pone.0048268.s001.tif]
