# Supplementary material for: Phylogeny-Based Comparative Methods Question the Adaptive Nature of Sporophytic Specializations in Mosses
Source: PLoS One. 2012 Oct 30;7(10):e48268. doi: 10.1371/journal.pone.0048268 (PMC3484137; doi:10.1371/journal.pone.0048268)
Supplement: Appendix S1 — Coding for habitat preferences and morphological character states. (DOC) [file pone.0048268.s002.doc]

Supporting information

Appendix S1. Coding for habitat preferences and morphological character states.

|  | Habitat preference | Post fertilization growth of perichaetial leaves | Operculum shape | **Dry peristome** | Spore size | **Basal membrane** | **Cilia** | Peristome | **Seta length** |
| --- | --- | --- | --- | --- | --- | --- | --- | --- | --- |
| Allaniella complanata | 1 | 1 | 0 | 1 | 1 | 1 | 1 | 1 | - |
| A. remota | 1 | 1 | 0 | 1 | 1 | - | 1 | 1 | 1 |
| A. urnigera | 1 | 1 | 0 | 1 | 1 | - | 1 | 1 | 1 |
| Bryolawtonia vancouveriensis | 1 | 1 | 0 | 0 | 0 | 0 | 0 | 0 | - |
| Camptochaete arbuscula | 1 | 1 | 0 | 0 | 0 | 0 | 0 | 0 | - |
| Circulifolium exiguum | 1 | 0 | 0 | 1 | 0 | - | 1 | 1 | 1 |
| C. microdendron | 1 | 0 | 0 | 1 | 0 | - | 1 | 1 | 1 |
| Dacryophyllum falcifolium | 0 | - | - | - | - | - | - | - | - |
| Dolichomitriopsis diversiformis | 1 | 1 | 0 | 1 | 0 | - | 1 | - | - |
| Echinodiopsis umbrosum | 0 | 1 | 1 | - | 0 | 0 | 0 | 0 | 0 |
| Forsstroemia trichomitria | 1 | 1 | 0 | 1 | 1 | 1 | 1 | 1 | 1 |
| Heterocladium heteropterum | 0 | 1 | 0 | 1 | 0 | 0 | 0 | 0 | - |
| H. dimorphum | 0 | 1 | 0 | 0 | 0 | 0 | 0 | 0 | 0 |
| H. macounii | 0 | 1 | 0 | 0 | 0 | 0 | 0 | 0 | 0 |
| H. procurrens | 0 | 1 | 0 | 0 | 0 | 0 | 0 | 0 | 0 |
| Himantocladium plumula | 1 | 0 | 0 | 1 | 1 | - | 1 | 1 | 1 |
| Homalia lusitanica | 0 | 0 | 0 | 0 | 0 | 0 | 0 | 0 | 0 |
| H. trichomanoides | 1 | 0 | 1 | 1 | 0 | 0 | 0 | 0 | 0 |
| H. webbiana | 0 | 0 | 0 | - | 0 | 0 | 0 | 0 | 0 |
| Isothecium myosuroides | 0 | 1 | 0 | 0 | 1 | 0 | 0 | 0 | 0 |
| Lembophyllum clandestinum | 1 | 1 | 1 | 0 | 0 | 0 | - | 0 | 0 |
| Leptodon smithii | 1 | 1 | 0 | 1 | 1 | 1 | 1 | 1 | 1 |
| L. longisetus | 1 | 1 | 0 | 1 | 1 | - | 1 | 1 | - |
| Neckera pennata | 1 | 1 | 0 | 1 | 1 | 1 | 1 | 1 | 1 |
| Neckeropsis calcicola | 0 | 0 | 0 | 0 | 1 | - | 1 | 1 | 1 |
| N. calcutensis | 0 | 1 | - | 1 | 1 | - | 1 | 1 | 1 |
| N. nitidula | 1 | 1 | 0 | 1 | 1 | - | 1 | 1 | 1 |
| Pendulothecium punctatum | 0 | 1 | 0 | 0 | 1 | - | - | 0 | 0 |
| Pinnatella alopecuroides | 1 | 0 | - | 1 | 1 | - | 1 | 1 | 1 |
| P. minuta | 1 | 0 | 0 | 1 | 0 | - | 1 | 1 | 1 |
| Porotrichodendron superbum | 1 | 1 | 1 | 1 | 0 | 0 | 1 | - | 0 |
| Porotrichum bigelovii | 0 | 1 | 1 | 1 | 0 | 0 | 0 | 0 | 0 |
| P. frahmii | 1 | 1 | 1 | 0 | 1 | - | 0 | 0 | 0 |
| P. fruticosum | 0 | 1 | - | 1 | 0 | 0 | 1 | - | 0 |
| Rigodium implexum | 0 | 1 | 0 | 0 | 0 | 0 | 0 | 0 | 0 |
| R. pseudothuidium | 1 | 1 | 0 | - | 0 | 0 | - | 0 | 0 |
| Taiwanobryum mucronata | 1 | 0 | - | 1 | 0 | - | 1 | 1 | - |
| T. speciosum | 1 | 1 | - | 1 | 1 | 1 | 1 | 1 | 0 |
| Thamnobryum alopecurum | 0 | 1 | 1 | 1 | 0 | 0 | 0 | 0 | 0 |
| T. maderense | 0 | 1 | 1 | 0 | 0 | 0 | 0 | 0 | 0 |
| T. subserratum | 0 | 1 | 1 | 1 | 0 | 0 | 0 | 0 | 0 |
| Thamnomalia glabella | 0 | 1 | 1 | 1 | 0 | - | 0 | 0 | 0 |
| T. tumidicaulis | 0 | - | - | - | - | - | - | - | - |
| Tripterocladium leucocladulum | 0 | 1 | 0 | - | 0 | 0 | 0 | 0 | 0 |
| Weymouthia mollis | 1 | 1 | 0 | - | 1 | 0 | - | 0 | 1 |

Character states for eight morphological traits and habitat preference were scored with binary coding using following delimitation for character states:

Habitat preference: on soil, humus or rocks (0); epiphyte or on exposed rocks or cliffs (1).

1. Post-fertilization growth of inner perichaetial leaves absent (0); or present (1).
2. Operculum shape conical or short-rostrate to rostrate (0); or long rostrate (1).
3. Dry peristome erect to spreading (0); or curved inwards to closed (1).
4. Spore size (median values) ≤ 16 µm (0); or > 16 µm (1).
5. Endostome basal membrane height > 30% of endostome (0); or absent or rudimentary (1). When the peristome height was < 30% of endostome, this intermediate form was coded as non-applicable (in *Dolichomitriopsis diversiformis*, *Porotrichodendron superbum,* and *Porotrichum fruticosum*).
6. Cilia well-developed. > 75% of segment length (0); or absent or rudimentary (1). Short cilia in *Lembophyllum clandestinum*, *Pendulothecium punctatum*, *Rigodium pseudothuidium,* and *Weymouthia mollis* are coded as non-applicable.
7. Peristome perfect (0); or reduced (1). Intermediate forms in *Dolichomitriopsis diversiformis*, *Porotrichodendron superbum,* and *Porotrichum fruticosum* are coded as non-applicable.
8. Seta length (median values) more than 9 mm (0); or < 3.5 mm (1). Intermediate forms in *Bryolawtonia vancouveriensis*, *Camptochaete arbuscula,* and *Leptodon longisetus* are coded as non-applicable.
