# Supplementary material for: Phylogeny-Based Comparative Methods Question the Adaptive Nature of Sporophytic Specializations in Mosses
Source: PLoS One. 2012 Oct 30;7(10):e48268. doi: 10.1371/journal.pone.0048268 (PMC3484137; doi:10.1371/journal.pone.0048268)
Supplement: Appendix S2 — Ancestral character state reconstructions for evolution of eight morphological characters and habitat preferences in the moss families Neckeraceae and Lembophyllaceae. (DOC) [file pone.0048268.s003.doc]

Supplementary material

Appendix 2. Ancestral character state reconstructions for evolution of eight morphological characters and habitat preferences in the moss families Neckeraceae and Lembophyllaceae.

|  | Node | **Habitat** | | 1) Post-fertilization | | 2) Operculum | | **3) Dry peristome** | | 4) Spore size | | **5) Basal membrane** | | **6) Cilia** | | 7) Peristome | | **8) Seta length** | | |
| --- | --- | --- | --- | --- | --- | --- | --- | --- | --- | --- | --- | --- | --- | --- | --- | --- | --- | --- | --- | --- |
|  |  | mean | stdev | mean | stdev | mean | stdev | mean | stdev | mean | stdev | mean | stdev | mean | stdev | mean | stdev | mean | stdev |  |
|  | 44 | **0.91** | 0.04 | 0.02 | 0.03 | 0.09 | 0.05 | **0.87** | 0.05 | 0.11 | 0.06 | 0.50 | 0.00 | **0.97** | 0.02 | **0.99** | 0.01 | **0.96** | 0.03 |  |
|  | 43 | **0.95** | 0.03 | 0.01 | 0.02 | 0.06 | 0.04 | **0.91** | 0.04 | 0.33 | 0.10 | 0.50 | 0.00 | **0.99** | 0.02 | **1.00** | 0.01 | **0.98** | 0.02 |  |
|  | 42 | **0.90** | 0.04 | 0.29 | 0.14 | 0.24 | 0.07 | 0.42 | 0.09 | **0.88** | 0.05 | 0.50 | 0.00 | **0.96** | 0.03 | **0.99** | 0.02 | **0.96** | 0.03 |  |
|  | 41 | **0.95** | 0.03 | 0.04 | 0.04 | 0.08 | 0.05 | **0.75** | 0.09 | **0.66** | 0.12 | 0.50 | 0.00 | **0.99** | 0.01 | **1.00** | 0.01 | **0.99** | 0.02 |  |
|  | 40 | **0.98** | 0.01 | 0.00 | 0.00 | 0.11 | 0.04 | **0.97** | 0.01 | **0.53** | 0.11 | 0.50 | 0.00 | **0.99** | 0.00 | **1.00** | 0.00 | **0.99** | 0.01 |  |
|  | 39 | **0.84** | 0.06 | 0.44 | 0.15 | 0.50 | 0.00 | **0.78** | 0.06 | **0.63** | 0.08 | **0.89** | 0.05 | **0.94** | 0.04 | **0.97** | 0.03 | 0.13 | 0.08 |  |
|  | 38 | **0.95** | 0.03 | 0.19 | 0.14 | 0.07 | 0.05 | **0.84** | 0.07 | **0.82** | 0.09 | 0.50 | 0.00 | **0.99** | 0.01 | **0.99** | 0.01 | **0.98** | 0.02 |  |
|  | 37 | **0.89** | 0.06 | 0.17 | 0.13 | 0.33 | 0.08 | **0.81** | 0.07 | **0.61** | 0.11 | **0.87** | 0.06 | **0.96** | 0.03 | **0.99** | 0.02 | 0.38 | 0.18 |  |
| **node E** | 36 | **0.92** | 0.05 | 0.13 | 0.14 | 0.15 | 0.08 | **0.83** | 0.07 | **0.75** | 0.13 | **0.82** | 0.09 | **0.98** | 0.02 | **0.99** | 0.01 | **0.79** | 0.18 |  |
|  | 35 | 0.28 | 0.17 | 0.36 | 0.25 | 0.19 | 0.09 | **0.92** | 0.04 | 0.40 | 0.15 | 0.15 | 0.16 | **0.99** | 0.01 | **0.97** | 0.02 | 0.30 | 0.22 |  |
|  | 34 | 0.18 | 0.12 | 0.04 | 0.04 | 0.50 | 0.13 | 0.34 | 0.08 | 0.16 | 0.07 | 0.03 | 0.05 | 0.00 | 0.02 | 0.00 | 0.01 | 0.04 | 0.05 |  |
|  | 33 | 0.01 | 0.01 | 0.97 | 0.02 | 0.49 | 0.15 | 0.10 | 0.04 | **0.63** | 0.10 | 0.05 | 0.07 | 0.01 | 0.03 | 0.00 | 0.00 | 0.01 | 0.01 |  |
| **node D** | 32 | **0.91** | 0.04 | **0.90** | 0.05 | **0.96** | 0.03 | 0.43 | 0.09 | 0.49 | 0.09 | 0.07 | 0.08 | 0.07 | 0.16 | 0.02 | 0.03 | 0.03 | 0.04 |  |
|  | 31 | 0.09 | 0.07 | **0.95** | 0.03 | **0.81** | 0.13 | 0.15 | 0.07 | **0.59** | 0.14 | 0.03 | 0.05 | 0.01 | 0.04 | 0.00 | 0.00 | 0.01 | 0.02 |  |
|  | 30 | 0.00 | 0.00 | **0.99** | 0.00 | **1.00** | 0.00 | 0.32 | 0.10 | 0.01 | 0.00 | 0.00 | 0.00 | 0.00 | 0.00 | 0.00 | 0.00 | 0.00 | 0.00 |  |
|  | 29 | 0.02 | 0.03 | **0.96** | 0.03 | **0.94** | 0.07 | 0.39 | 0.13 | 0.33 | 0.14 | 0.01 | 0.03 | 0.00 | 0.01 | 0.00 | 0.00 | 0.01 | 0.02 |  |
|  | 28 | 0.01 | 0.02 | **0.93** | 0.04 | **0.97** | 0.02 | **0.62** | 0.11 | 0.08 | 0.04 | 0.01 | 0.03 | 0.00 | 0.01 | 0.00 | 0.00 | 0.02 | 0.03 |  |
|  | 27 | 0.14 | 0.15 | 0.09 | 0.12 | 0.26 | 0.14 | **0.72** | 0.11 | 0.18 | 0.10 | 0.03 | 0.06 | 0.07 | 0.17 | 0.08 | 0.16 | 0.07 | 0.10 |  |
|  | 26 | 0.01 | 0.02 | **0.94** | 0.04 | **0.97** | 0.04 | 0.49 | 0.16 | 0.18 | 0.10 | 0.01 | 0.03 | 0.00 | 0.01 | 0.00 | 0.00 | 0.01 | 0.02 |  |
|  | 25 | **0.97** | 0.02 | **0.96** | 0.02 | 0.03 | 0.02 | **0.95** | 0.02 | **0.97** | 0.02 | 0.50 | 0.00 | **0.99** | 0.01 | **1.00** | 0.00 | **0.99** | 0.01 |  |
|  | 24 | **0.98** | 0.01 | **0.98** | 0.01 | 0.02 | 0.02 | **0.97** | 0.01 | **0.98** | 0.01 | **0.93** | 0.04 | **0.99** | 0.01 | **1.00** | 0.00 | **0.96** | 0.02 |  |
|  | 23 | **0.99** | 0.00 | **0.99** | 0.00 | 0.01 | 0.00 | **0.99** | 0.00 | **0.99** | 0.00 | **0.98** | 0.01 | **1.00** | 0.00 | **1.00** | 0.00 | **0.97** | 0.02 |  |
|  | 22 | **0.98** | 0.01 | **0.98** | 0.02 | 0.02 | 0.02 | **0.96** | 0.02 | **0.98** | 0.02 | **0.99** | 0.02 | **0.99** | 0.01 | **1.00** | 0.00 | **0.98** | 0.01 |  |
|  | 21 | **0.97** | 0.02 | **0.97** | 0.02 | 0.03 | 0.02 | **0.95** | 0.02 | **0.97** | 0.02 | **0.99** | 0.01 | **0.99** | 0.01 | **1.00** | 0.00 | **0.99** | 0.01 |  |
| **node C** | 20 | **0.95** | 0.02 | **0.95** | 0.03 | 0.05 | 0.03 | **0.93** | 0.03 | **0.95** | 0.03 | **0.99** | 0.01 | **0.99** | 0.01 | **0.99** | 0.01 | **0.98** | 0.02 |  |
|  | 19 | 0.01 | 0.02 | **0.77** | 0.06 | **0.85** | 0.06 | **0.73** | 0.05 | 0.24 | 0.06 | 0.50 | 0.00 | 0.02 | 0.04 | 0.01 | 0.03 | 0.11 | 0.08 |  |
|  | 18 | 0.02 | 0.05 | 0.29 | 0.25 | **0.73** | 0.19 | **0.66** | 0.19 | 0.11 | 0.09 | 0.01 | 0.02 | 0.01 | 0.04 | 0.01 | 0.02 | 0.02 | 0.04 |  |
|  | 17 | 0.24 | 0.15 | **0.86** | 0.06 | 0.44 | 0.13 | **0.81** | 0.06 | **0.64** | 0.08 | **0.91** | 0.05 | 0.10 | 0.22 | 0.15 | 0.28 | **0.54** | 0.20 |  |
| **node I** | 16 | 0.07 | 0.10 | **0.57** | 0.25 | **0.60** | 0.23 | **0.75** | 0.13 | 0.37 | 0.14 | 0.25 | 0.25 | 0.03 | 0.09 | 0.03 | 0.08 | 0.17 | 0.16 |  |
|  | 15 | **0.98** | 0.01 | **0.98** | 0.01 | 0.03 | 0.05 | 0.08 | 0.03 | 0.35 | 0.11 | 0.00 | 0.01 | 0.01 | 0.02 | 0.00 | 0.00 | **0.88** | 0.12 |  |
| **node B** | 14 | **0.98** | 0.01 | **0.98** | 0.01 | 0.38 | 0.16 | 0.03 | 0.02 | 0.13 | 0.07 | 0.00 | 0.01 | 0.01 | 0.02 | 0.00 | 0.00 | 0.36 | 0.18 |  |
|  | 13 | 0.21 | 0.15 | **1.00** | 0.00 | 0.00 | 0.00 | 0.05 | 0.02 | 0.00 | 0.00 | 0.00 | 0.00 | 0.00 | 0.01 | 0.00 | 0.00 | 0.00 | 0.00 |  |
|  | 12 | **0.58** | 0.23 | **0.95** | 0.03 | 0.15 | 0.10 | 0.05 | 0.03 | 0.08 | 0.05 | 0.01 | 0.02 | 0.00 | 0.01 | 0.00 | 0.00 | 0.08 | 0.09 |  |
|  | 11 | 0.01 | 0.02 | **0.94** | 0.03 | 0.05 | 0.03 | 0.25 | 0.07 | 0.29 | 0.08 | 0.01 | 0.02 | 0.00 | 0.01 | 0.00 | 0.00 | 0.02 | 0.02 |  |
| **node A** | 10 | **0.91** | 0.04 | **0.90** | 0.05 | 0.10 | 0.06 | 0.41 | 0.09 | 0.12 | 0.06 | 0.07 | 0.09 | **0.93** | 0.15 | 0.02 | 0.03 | 0.50 | 0.00 |  |
|  | 9 | 0.10 | 0.09 | **0.97** | 0.02 | 0.03 | 0.02 | 0.23 | 0.10 | 0.12 | 0.06 | 0.01 | 0.02 | 0.00 | 0.01 | 0.00 | 0.00 | 0.04 | 0.04 |  |
|  | 8 | 0.23 | 0.22 | **0.98** | 0.01 | 0.04 | 0.03 | 0.05 | 0.04 | 0.05 | 0.03 | 0.00 | 0.01 | 0.00 | 0.00 | 0.00 | 0.00 | 0.02 | 0.03 |  |
|  | 7 | 0.01 | 0.01 | **0.95** | 0.03 | 0.05 | 0.03 | **0.53** | 0.11 | 0.06 | 0.03 | 0.01 | 0.02 | 0.00 | 0.01 | 0.00 | 0.00 | 0.10 | 0.07 |  |
|  | 6 | 0.08 | 0.12 | **0.90** | 0.05 | 0.10 | 0.06 | 0.22 | 0.10 | 0.13 | 0.07 | 0.02 | 0.04 | 0.00 | 0.01 | 0.00 | 0.01 | 0.05 | 0.06 |  |
| **node II** | 5 | 0.04 | 0.09 | **0.76** | 0.20 | 0.33 | 0.22 | **0.51** | 0.18 | 0.19 | 0.11 | 0.06 | 0.11 | 0.01 | 0.03 | 0.00 | 0.02 | 0.06 | 0.09 |  |
|  | 4 | 0.00 | 0.00 | **0.99** | 0.00 | 0.01 | 0.00 | 0.01 | 0.00 | 0.01 | 0.00 | 0.00 | 0.00 | 0.00 | 0.00 | 0.00 | 0.00 | 0.00 | 0.00 |  |
|  | 3 | 0.02 | 0.05 | **0.85** | 0.15 | 0.21 | 0.17 | 0.30 | 0.16 | 0.13 | 0.09 | 0.03 | 0.07 | 0.00 | 0.01 | 0.00 | 0.01 | 0.03 | 0.06 |  |
|  | 2 | 0.02 | 0.04 | **0.82** | 0.14 | 0.22 | 0.16 | 0.31 | 0.15 | 0.16 | 0.09 | 0.04 | 0.08 | 0.00 | 0.02 | 0.00 | 0.01 | 0.04 | 0.06 |  |
|  | 1 | 0.00 | 0.00 | 0.00 | 0.00 | 0.00 | 0.00 | 0.44 | 0.08 | 0.00 | 0.00 | 0.00 | 0.00 | 0.00 | 0.00 | 0.00 | 0.00 | 0.00 | 0.00 |  |

Results for ancestral character state reconstructions for evolution of eight morphological characters and habitat preferences in the moss families Neckeraceae and Lembophyllaceae using a Bayesian approach in the program BayesTraits (Pagel & Meade 2004). Probabilities for derived character state (P(1)) is given, and favored character state at the node is indicated in bold. Numbering for the nodes in the phylogeny is in the Supplementary material Fig. S1. All characters have binary coding. Nodes A to E show a shift in habitats from soil, humus or rocks to epiphytism or to exposed rocks or cliffs. Node I is the ancestor of Neckeraceae and Lembophyllaceae, and node II the ancestor of all Neckeraceae species
